# Application of SPECT and PET / CT with computer-aided diagnosis in bone metastasis of prostate cancer: a review

**DOI:** 10.1186/s40644-022-00456-4

**Published:** 2022-04-15

**Authors:** Zhao Chen, Xueqi Chen, Rongfu Wang

**Affiliations:** 1 Department of Nuclear Medicine, Peking University First Hospital, Xicheng District, Beijing, 100034 China; 2 Department of Nuclear Medicine, Peking University International Hospital, Changping District, Beijing, 102206 China

**Keywords:** SPECT, PET/CT, Computer-aided diagnosis, Bone metastasis, Prostate cancer

## Abstract

Bone metastasis has a significant influence on the prognosis of prostate cancer(PCa) patients. In this review, we discussed the current application of PCa bone metastasis diagnosis with single-photon emission computed tomography (SPECT) and positron emission tomography/computed tomography (PET/CT) computer-aided diagnosis(CAD) systems. A literature search identified articles concentrated on PCa bone metastasis and PET/CT or SPECT CAD systems using the PubMed database. We summarized the previous studies focused on CAD systems and manual quantitative markers calculation, and the coincidence rate was acceptable. We also analyzed the quantification methods, advantages, and disadvantages of CAD systems. CAD systems can detect abnormal lesions of PCa patients’ ^99m^Tc-MDP-SPECT, ^18^F-FDG-PET/CT, ^18^F-NaF-PET/CT, and ^68^ Ga-PSMA PET/CT images automated or semi-automated. CAD systems can also calculate the quantitative markers, which can quantify PCa patients’ whole-body bone metastasis tumor burden accurately and quickly and give a standardized and objective result. SPECT and PET/CT CAD systems are potential tools to monitor and quantify bone metastasis lesions of PCa patients simply and accurately, the future clinical application of CAD systems in diagnosing PCa bone metastasis lesions is necessary and feasible.

## Introduction

Currently, prostate cancer (PCa) patients are the most common and the second leading death cancer among men in the United States; luckily, PCa survival is highest [1]. However, more than 90% of PCa patients could develop bone metastases [2], which may significantly reduce the survival time and affect the treatment [3]. Therefore, detecting metastases at an early phase of PCa is essential [4].

The PCa bone metastasis lesions progression and reactive sclerosis after treatment show similar performance on CT images, both high-density lesions. Due to the osteoblastic feature of PCa bone metastasis [5], the RECIST 1.1 criteria proposed it as immeasurable lesions [6].

As a functional imaging technique, nuclear medicine imaging shows physiological processes, which may detect bone metastasis lesions earlier than CT and MRI, as the anatomical changes of bone lesions always lag behind the functional changes. Bone scintigraphy has been used for the detection and evaluation of bone metastasis lesions for many years, because it can evaluate the whole skeleton sensitively, quickly, and cheaply [7]. Another commonly used method to detect bone metastasis lesions is PET/CT, which has better image quality than bone scintigraphy and gives more information besides bone metastasis.

However, the clinicians always need to spend much time identifying bone metastasis lesions on SPECT or PET/CT images, even though the results lack quantitative diagnosis. The accuracy and sensitivity of defining bone metastasis lesions are relatively subjective and only depend on the experience of clinicians. In a study, the sensitivities of 37 clinical readers ranged from 52 to 100% [8]. In another survey, the kappa agreement among readers varied from 0.16 to 0.82 [9]. CAD analyzes some data in case samples by developing various image processing algorithms and deep learning [10], and then develops a model to associate the extracted information with specific disease results. CAD is a tool rather than a doctor’s replacement, which is different from automated computer diagnosis.

In recent years, artificial neural network (ANN) and convolutional Neural Networks (CNN) are commonly used deep learning models in medical image analysis [11, 12]. Besides gradient descent and backpropagation of ANN, CNN has an additional set and convolution layer. Nowadays, CNNs methods for medical images is widely be used, such as Xception [13]. Generally, CAD in medical imaging is divided into three steps [14]: the first step is to extract the lesions from the normal structure; The second step is the quantization of image features; The third step is to process the data and draw a conclusion. Because the computer can make full use of image information for accurate quantitative calculation without human subjectivity, and avoid different diagnostic results caused by different personal knowledge and experience; Therefore, its result is unambiguous and definite. It makes the diagnosis more accurate and scientific. To improve the stability and sensitivity of lesions evaluation, CAD systems for bone scintigraphy or PET/CT images have been developed and put into use [15].

### Evidence acquisition

PubMed search of the English literature was performed using the following terms: 'bone scan', 'bone scintigraphy', 'SPECT', 'PET/CT', 'prostate cancer', 'bone metastasis', 'computer-aided diagnose', 'automatic', 'deep learning'. The articles searched from PubMed and its reference list of the articles were reviewed. The studies concentrated on the relevant theme to this manuscript were synthesized by the first author. Then a first draft was prepared and distributed to the corresponding author for critical review. After several iterations, a consensus was reached on the manuscript’s content and structure and submitted here.

### SPECT

The principle of bone scintigraphy is the high uptake of technetium-99 m methylenediphosphonate (^99m^Tc-MDP), a bone-metabolism radionuclide other than a tumor-specific radionuclide, at the sites of bone repair where maybe the bone metastasis lesions. At 3 h post-injection, only 3%-5% of ^99m^Tc-MDP distributed in the blood [16], which makes the abnormal bone lesions easier to be detected.

Bone scan index (BSI) was proposed to quantify bone scintigraphy images [17]. However, the manual process of BSI calculation could take much time and the mistakes are hard to avoid. BSI calculation with CAD systems is fast, objective, and precise. It is necessary to apply CAD systems to quantify bone scintigraphy.

#### Bone scintigraphy quantification methods

Over the years, the semi-automatic bone scintigraphy images segmentation method [18], the characteristic point-based algorithm [19], the adaptive thresholding with different cut-offs segmentation algorithm [20], the temporal subtraction-based interval change detection algorithm [21], the features derived classification method [22] and the active shape model segmentation [15] were developed to quantify bone scintigraphy images. Nevertheless, these methods are time-consuming and their accuracy is still low [23]. The possible reasons may be that they are sensitive to image noise and independently process anterior and posterior images. U-Net-type convolutional networks are applied to quantify bone metastasis, which may contribute to detecting bone metastasis lesions more accurately than before [24]. The butterfly-type network (BtrflyNet) can process anterior and posterior bone scintigraphy images with two U-Nets at the same time [25]. Shimizu et al. [26] proposed that BtrflyNet combined with deep supervision (DSV) and residual learning is the most accurate system.

Currently, the widely used software package for calculating BSI is BONENAVI (FUJIFILM Toyama Chemical, Co. Ltd, Tokyo, Japan) and EXINI bone (EXINI Diagnostics AB, Lund, Sweden), which can identify, quantify and diagnose bone lesions. The detection sensitivity and specificity of bone metastasis lesions are almost 90% [15] and the correlation with manual calculated BSI is 0.80 [27]. The software was based on ANN technology and Morphon registration, which can accord the in-built skeleton atlas to the anterior and posterior images [28]. All hot spots on anterior and posterior images were detected independently in the atlas. Therefore, ANN, ranging from 0 (non-metastatic) to 1 (metastatic), was quantified on each hot spot. Then, BSI was calculated on each spot with ANN > 0.5. In recent years, some new procedures [29, 30] have shown good performance in automatic identification of bone metastasis lesions in the bone scintigraphy images. Research [31] also proposed a CNN that can diagnose bone metastasis lesions automatically in bone scintigraphy images for the first time.

BSI could change over time, because of the dynamic ^99m^Tc-MDP metabolism in the body, especially in pelvic and spine abnormal lesions. Recent literature [10] has confirmed that deep learning has high accuracy in identifying bone metastasis lesions of PCa. It is necessary to take bone scintigraphy regularly and calculate BSI accurately. Only by this way, BSI can be a quantitative marker to monitor prostate patients’ bone metastasis dynamic changes.

#### Clinical application and advantages of bone scintigraphy CAD systems in PCa

The quantitative markers to judge bone lesions whether metastases are ANN value and BSI, which are analyzed by per region or per patient.

ANN value is related to the bone metastasis possibility, which is a good parameter to detect bone metastasis [32]. There are two types of ANN value: region-based ANN value and patient-based ANN value [33]. Both of them range from 0 to 1. Region-based ANN value range between 0.5 and 1 means the bone lesion is suspected to metastasize, and patient-based ANN value range between 0.5 and 1 means the case is considered to have bone metastasis. When ANN value range from 0 to 0.5, the conclusion converses.

BSI is indicated to the bone metastasis extent of PCa patients, which may be associated with the progression, remission [34], and prognosis [27] of PCa patients. BSI is the percentage of the suspected bone metastasis lesions total count to that of the whole skeleton. BSI > 0 means the patient probably has done bone metastasis [35]. BSI > 1 indicates the 5-year probability of survival is very low, even 0% for those BSI > 5 [36]. The diagnostic bone metastasis accuracy of BSI is based on ANN. A study [37] reported that BSI may be better than TLG in predicting the prognosis of patients, CAD systems can also quantify potential metastatic lesions from multiple bone scintigraphy images of a patient, which is of great significance for evaluating the condition of patients, adjusting treatment plans and predicting the prognosis.

The bone metastasis of PCa is almost osteoblastic [5], which is easy to be detected by bone scintigraphy. The sensitivity of bone scintigraphy is high and the bone metastasis lesions could be discovered earlier than skeletal radiograms. Tokuda et al. [38] reported CAD systems' positive predictive value of bone metastasis of the PCa group was much higher than other cancer groups. Takuro et al. [39] advanced the ANN of the PCa group were the highest in their survey, which means the accuracy of BSI is acceptable in PCa patients. BSI has been proposed to improve PCa patients' bone metastasis diagnostic accuracy [40]. The deep learning model has good accuracy in identifying metastatic lesions independently [10, 41].

Moreover, compared with manual BSI calculation, the deep learning model takes less time to calculate BSI and has better repeatability [31, 34, 42, 43]. According to literature reports [15, 33], some CAD systems have been applied in clinical practice, but they have not been used on a large scale. A study [37] reported that BSI may be better than TLG in predicting the prognosis of patients. CAD systems can also quantify potential metastatic lesions from multiple bone scans, which is of great significance for evaluating the condition of patients, adjusting treatment plans, and predicting the prognosis. Therefore, bone scintigraphy CAD systems may have great potential application value in PCa patients with bone metastasis.

#### Limitation of bone scintigraphy CAD systems in PCa

At the process of hot spot detection, bone and other organs' normal high uptake sites may be detected as bone lesions, such as bladder, and kidneys. 5% of patients' automated detection needs to be manually corrected [27].

Even for typical bone metastasis lesions, the diagnosis criteria of BSI > 0 had a low specificity and positive predictive value, and the diagnosis criteria of BSI > 1 had low sensitivity [44].

### PET/CT

The commonly used radionuclides for detecting PCa bone metastasis lesions are radionuclide fluorine-18-fluorodeoxyglucose(^18^F-FDG), gallium 68 prostate-specific membrane antigen (^68^ Ga-PSMA) and fluorine 18 sodium fluoride(^18^F-NaF). ^68^ Ga-PSMA is a tumor-specific radioactive probe. The expression of PSMA in normal prostate tissue is low, but in PCa and metastasis lesions, PSMA expression is 1000 times higher than normal tissue [45]. ^18^F-NaF is similar to ^99m^Tc-MDP, which can reflect the repair of bone. But the widely used PET/CT ^18^F-FDG shows limited value in PCa because most PCa bone metastasis lesions have low glycolytic rates [46].

Due to more information given by PET/CT, readers need more time to assess every possible metastasis lesion. Furthermore, visual assessment of PET/CT images is always based on every lesion independently, and the standard uptake values(SUV) of every single lesion cannot reflect the general condition of the patient. Nowadays, metabolic tumor volume(MTV) and total lesion glycolysis(TLG) are widely used as PET/CT quantitative markers to assess whole-body tumor burden. Besides, manual whole-body quantification is time-consuming. It is essential to develop CAD systems for quantifying whole-body tumor burden. Previous studies [47] verified that the manual and semi-automatic quantification were highly consistent. The step of using CAD system for PET / CT images [14] is to segment and extract the features of CT images and PET images respectively. After extracting features from CT and PET images, they are sent to the classification respectively. If necessary, the PET/CT images will be sent to the mixed classification.

#### PET/CT image quantification methods

In the CAD system, the development of quantification system is very important. In the past few years, ^18^F-FDG-PET/CT semi-automated quantify system PERCIST and ^18^F-NaF-PET/CT semi-automated system proposed by Etchebere et al. [48] showed limited value in the application of PCa bone metastasis lesions assessment. Currently, semi-automatic [49] and automated [50] bone metastasis lesions quantify systems were proposed based on ^68^ Ga-PSMA-PET/CT. Gafita et al. developed a whole-body semi-automatic system named aPSMA based on the original semi-automatic system, which can assess the lymph node and organ metastasis condition besides bone [51].

Quantitative markers of ^68^ Ga-PSMA-PET/CT were proposed by imitating the calculation of BSI [49], MTV, and TLG [52]. The process of quantitative markers calculation is as follows: Firstly, CT and PET images are to be read by the system. The segmentation of CT images and PET images are independent, with CT images used for locating and PET images used for detecting bone lesions. Global thresholding, local thresholding, and morphological hole closing are used to segment the bone mask from CT images. The high uptake sites where SUV values are above the threshold are supposed as bone metastasis lesions in PET images. The way of distinguishing bone metastasis lesions by SUV values has high accuracy, as ^68^ Ga-PSMA is a tumor-specific radionuclide. The threshold is chosen by analyzing PET images of the patients without bone metastasis lesions. Finally, quantitative markers calculation is based on the tumor volume and SUV values segmented by the system.

#### Clinical application and advantages of PET/CT CAD systems in PCa

Currently, whole-body total-lesion PSMA(TL-PSMA) and whole-body PSMA-tumor volume(PSMA-TV) are commonly applied as ^68^ Ga-PSMA-PET/CT quantitative markers. Whole-body PSMA-TV is the percentage of the suspected bone metastasis lesions total volume to that of the whole skeleton. Whole-body TL-PSMA is the sum of all bone metastasis lesions volume and SUVmean values multiplication. The quantitative markers show higher concordance with Gleason score results [53] and PSA levels [54]. The higher the values of the quantitative markers means the worse the prognosis.

Different segmentation and correction methods will not affect the diagnostic and predictive value of PET/CT [55]. The quantification markers of PET/CT have good repeatability and stability.

Pyka et al. [56] proposed the bone metastasis lesions diagnostic accuracy, sensitivity, and specificity of ^68^ Ga-PSMA-PET/CT are better than bone scintigraphy. Because bone scintigraphy shows the bone reactive changes and ^68^ Ga-PSMA-PET/CT shows the specific PSMA expression on bone metastasis lesions. CAD systems calculate quantitative markers only need a few minutes, even the manual correction is very simple. And correction time required could be further reduced by some machine training [27]. By contrast, the manual whole-body tumor burden assessment required several hours. Quantitative markers had the consistency of PSV levels, BSI, and PERCIST results, and the changing trend of quantitative markers is different from SUV values in the treatment process [57], which may provide some information undiscovered yet.

#### Limitation of PET/CT CAD systems in PCa

Commonly, the PET/CT scan range does not include arms and legs. To standardize the calculation of BSIs, CAD systems only detect the range from the first thoracic vertebra to the bottom of the ischium, and the metastasis lesions on the skull and bone of limbs are ignored by CAD systems.

The manual correction of CT image segmentation is required when calcification is heavy or artifact is existing, and the misalignment of ribs on CT and PET images needs to be corrected due to lack of respiratory gating devices [49]. Because of respiration or movement, a bone mask segmented from CT images may exclude some bone lesions, which may be regarded as extra-bone metastasis lesions by CAD systems. Nowadays, an algorithm has been developed to correct these problems [51]. Some normal uptake soft tissue may be misdiagnosed as bone lesions by CAD systems when CT and PET images alignment was not accurate. A visual inspection is essential for CAD systems results.

Some benign bone lesions could also uptake ^68^ Ga-PSMA at a high level, such as fracture repair, degenerative osteoarthropathy, and osteitis deformans [58]. Manual combination with CT images is necessary to improve the accuracy of CAD systems results. It is difficult to judge whether a lesion is benign or malignant when ^68^ Ga-PSMA PET shows high uptake but CT density and morphological features are normal.

## Discussion

Bone metastasis lesions are not demonstrated in the same way on bone scintigraphy. Some uncommon bone lesions should be diagnosed by experienced doctors, and CAD systems may give the wrong result. For example, in some special super bone scan images, CAD systems may ignore the whole skeleton’s extremely average and symmetrical metastasis lesions, because no hot spot could be detected. However, experienced nuclear medicine doctors would diagnose bone metastasis by 'absent kidney sign' and a combination of PSA levels, CT, MRI, and clinical features. There are little research on the abnormal bone metastasis lesions diagnostic accuracy rate of CAD systems yet.

The bone scintigraphy and PET/CT have limited application value in bone metastasis lesions after treatment because of the flare effect and reactive sclerosis. The sensitivity of bone scintigraphy is low in patients with low PSA values [59]. Currently, SPECT and PET/CT are still considered as the gold standard of bone metastasis, as tissue biopsy is hard to accomplish.

The SPECT and PET/CT quantitative markers performed well on the assessment of whole-body bone tumor burden, but have still not been widely applied yet. The most important reason is that manual calculation is time-consuming, which limits the application of patients with multiple bone metastases. CAD systems increase the clinical application possibilities of whole-body tumor burden assessment. PET/CT may be wider used when it could be covered by medical insurance reimbursement.

## Conclusion

SPECT and PET/CT, as multiple molecular imaging techniques, with CAD systems are simple and accurate tools to assess PCa patients’ whole-body bone tumor burden, which is important to predict prognosis and guide treatment of patients. Nowadays, the application of CAD systems is still limited to experimental research. Considerable research will be needed to assess the sensitivity, specificity, and diagnostic accuracy of CAD systems and support the clinical application value of SPECT and PET/CT CAD systems.

## Data Availability

Not applicable.

## Abbreviations

PCa: Prostate cancer
SPECT: Single-photon emission computed tomography
PET/CT: Positron emission tomography/computed tomography
CAD: Computer-aided diagnosis
ANN: Artificial neural network
CNN: Convolutional Neural Networks
^99m^Tc-MDP: Technetium-99 m methylenediphosphonate
BSI: Bone scan index
BtrflyNet: Butterfly-type network
DSV: Deep supervision
^18^F-FDG: Fluorine-18-fluorodeoxyglucose
^68^ Ga-PSMA: Gallium 68 prostate-specific membrane antigen
^18^F-NaF: Fluorine 18 sodium fluoride
SUV: Standard uptake values
MTV: Metabolic tumor volume
TLG: Total lesion glycolysis
TL-PSMA: Total-lesion PSMA
PSMA-TV: PSMA-tumor volume
